# ﻿Description and life history of a new genus and species of Limacodidae (Lepidoptera, Zygaenoidea) from Taiwan, the first with a monkey-slug-like caterpillar from outside the New World

**DOI:** 10.3897/zookeys.1261.155406

**Published:** 2025-11-21

**Authors:** Yu-Chi Lin, Rung-Juen Lin, Marc E. Epstein

**Affiliations:** 1 Department of Life Science, National Taiwan Normal University, Tingzhou Rd., Taipei 116059, Taiwan National Taiwan Normal University Taipei Taiwan; 2 Department of Pediatrics and Medical Genetics, National Taiwan University Hospital, Zhongshan S. Rd., Taipei 100226, Taiwan National Taiwan University Hospital Taipen Taiwan; 3 California Department of Food & Agriculture, 3294 Meadowview Rd., Sacramento, CA 95832-1448, USA California Department of Food & Agriculture Sacramento United States of America

**Keywords:** Detachable tubercles, hag moth, immature, mimicry, monkey slug, *
Phrixolepia
*, *
Yufengus
*

## Abstract

The genus *Yufengus***gen. nov.** is established for a new species of Limacodidae, *Y. atrophaneuroides***sp. nov.***Yufengus* is from Taiwan, yet its larva is similar to monkey-slug caterpillars in the New World (*Phobetron* Hübner, 1825: Limacodidae) in being covered with hairy, detachable tubercles. Monkey-slug caterpillars are currently known to form a monophyletic group which include as many as eight genera in the Americas referred to as the *Phobetron* complex by Epstein (1996). Although the larva of *Yufengus* appears, in particular, most similar to the genus *Phobetron*, a closer look at morphology reveals fundamental differences that are shared with Asian *Phrixolepia* Butler, 1877. Caterpillars of the new genus are structurally similar to *Phrixolepia*, but *Phrixolepia* is more translucent, less hairy, and differs in other characters including those of adults, separated by wing pattern and genitalia. In addition to describing the new genus *Yufengus*, we explore whether it is indeed in the same clade as the *Phobetron* complex of genera from the New World. The life history and morphology of adults and immature stages are described in this study.

## ﻿Introduction

The slug caterpillar moths (Limacodidae) exhibit a remarkable diversity in larval form. Although all limacodid caterpillars have a slug-like ventral surface, the dorsa are classified into three main types: 1) the “Nettle slug” caterpillar, the most common, is armed with rows of spines; 2) the “Gelatine slug” caterpillar, a non-stinging type of limacodid larvae, has a relatively smooth surface; and 3) the slug caterpillar with fleshy, detachable outgrowths (= tubercles), the rarest, has many fine setae (Dyar 1896, 1907, 1914; Cock et al. 1987; Epstein 1996; Zaspel et al. 2016; Lin et al. 2019). Among the last group, one of the most bizarre and recognizable species is the hairy non-venomous “monkey-slug” caterpillar or “hag moth”, *Phobetron pithecium* (Smith, 1797), whose fluffy appearance results from its setae-covered detachable tubercles.

The New World genus *Isochaetes* Dyar, 1899 has similar but translucent tubercles, hence the common name “spun-glass-slug moth”, although other genera occur in both the Neotropics and parts of Asia (Epstein and Corrales 2004; Solovyev and Witt 2009; Lin et al. 2019). New World limacodid genera known to possess detachable tubercles all appear to be in the clade referred to as the *Phobetron* complex (Epstein 1996): *Phobetron* Hübner, 1825, *Isochaetes* Dyar, 1899, *Alarodia* Möschler, 1886, some species in *Euphobetron* Dyar, 1905, *Vipsophobetron* Dyar, 1905, *Microphobetron* Dyar, 1912, *Leucophobetron* Dyar, 1897, and *Heuretes* Grote & Robinson, 1868 (see review of *Heuretes*, *Alarodia*, and *Leucophobetron* in Epstein and Miller 1990). In Asia, *Phrixolepia* Butler, 1877 and *Olona* Snellen, 1900 are the only published examples of caterpillars with these detachable tubercles (Solovyev 2009a; Solovyev et al. 2012).

The only modern phylogenetic studies of Limacodidae that included more than one genus in the *Phobetron* complex are those works by Zaspel et al. (2016) and Epstein et al. (in press). Zaspel included *Phobetron pithecium* (Smith), *Isochaetes beutenmuelleri* (H. Edwards), *Vipsophobetron marisa* (Druce), and *Alarodia slossoniae* (Packard). Epstein et al. includes the same species, except *Phobetron hipparchia* (Cramer) rather than *P. pithecium*, and *Microphobetron* rather than *V. marisa.* In both studies, the *Phobetron* complex is a clade among the gelatine caterpillars. This is contrary to Dyar (1899), which had *Phobetron* as sister to the “spined Eucliids” now referred to as nettles. Curiously, Dyar was aware that *Phobetron* fed as first instars, unlike the nettles that fast (molt quickly without feeding) in their first instar, but he held the view that these were an early branch of nettles rather than a gelatine (see Zaspel et al. 2016).

Lin et al. (2019) indicated that (*Phrixolepia* + *Isochaetes*) + “sp. 2” (= *Yufengus atrophaneuroides*) were a monophyletic group within the gelatine group. Liang et al. (2024), which did not include New World taxa such as *Isochaetes*, found that *Phrixolepia + Pseudidonauton* Hering was sister to both the gelatine and nettle clades. Although the position of *Phrixolepia* + *Pseudodinauton* is different, this result showing *Pseudidonauton* to be sister to *Phrixolepia* suggests that it may be another group with deciduous tubercles.

Lin did not describe *Yufengus* in 2019 because its adults were known only from female samples. The larva and adult morphology of the new genus and species are fundamentally different from others known to have deciduous tubercles from Asia: *Phrixolepia*, *Olona*, and *Pseudidonauton* (Holloway 1986; Cock et al. 1987; Solovyev 2009a, 2009b; Solovyev et al. 2012; Wu et al. 2021).

Limacodid diversity in Taiwan is notably high, given the island’s relatively small land area. Over 30 years ago the family was known to be represented by 31 genera and 50 species, as documented by Inoue (1992) in *Lepidoptera of Taiwan*. Solovyev (2017) added six additional species to the fauna, increasing the total to 43 genera and 58 species. Prior to the addition of *Yufengus atrophaneuroides* sp. nov., according to the Catalogue of Life in Taiwan website, there were 44 genera and 62 species of Limacodidae recorded in Taiwan (Shao and Chung 2024).

Herein, we describe the new species in a new genus as well as its life history with a direction towards future phylogenetic studies that will need more taxon sampling to better understand the evolution of the unique larval characteristics found in *Yufengus*. This should elucidate whether deciduous tubercles in Asian and New World limacodids are ancestral or independently derived.

## ﻿Materials and methods

Late instars were collected in the field in New Taipei City from their larval host plants, typically on the abaxial surface of mature leaves. Eggs and early instars were obtained from a female that was collected at a mercury light trap in Hualien County. Larvae were reared individually in plastic containers (150 × 75 × 45 mm). Fresh foliage was provided with moist tissue paper to prevent dehydration. The rearing conditions were kept at a constant temperature of 25 °C and 16:8 [L:D] per day. Descriptions of the immature stages and observations on the species’ biology were based on the rearing lot HSU 16C67M, 22B39M, and 22B40M. The rearing lot follows the system of Powell and De Benedictis (1995), where “HSU 22B40M” refers to the name of the rearing database (HSU), the year (22 for 2022), the month (B for February), the sequential number of collection (15), and the taxonomic group (M for moth). Voucher material is deposited in the Department of Life Science, National Taiwan Normal University, Taipei, Taiwan (**NTNU**).

Scanning electron microscopy (SEM) of adult legs and caterpillars was performed with JEOL JSM-5600 scanning electron microscope. Following critical point drying (CPD), we attached the samples to stubs (15 mm diameter) and used a LADD critical-point dryer and coated them with gold palladium using a JEOL JEE-400 Vacuum Evaporator.

Wing venation was prepared by first removing wings with micro-scissors and forceps under the microscope. The wings were then moistened with 70% ethanol in a petri dish. A diluted bleach solution (ratio of bleach to water was one to four) was poured into the petri dish to soak the wing set for a few minutes, after which the scales of the wing were removed by a fine brush. Finally, the wing set was fixed in 95% ethanol and then 100% ethanol before being mounted in Euparal and preserved on glass microscope slides.

Dissection of the genitalia was performed by first removing the entire abdomen, which was placed in 10% KOH and boiled for 10 min. The abdominal integument and genital capsule stained in a weak solution of Phthalocyanine pigment for an hour, and then transferred to 30% ethanol for cleaning, dissection, and examination. Completed dissections were fixed in 95% ethanol and then 100% ethanol before being mounted in Euparal and preserved on glass microscope slides.

Morphological characters follow those in Epstein et al. (in press). Primary types are deposited in the following collections:

**BMNH** The Natural History Museum, London

**NTNU** Department of Biology, National Taiwan Normal University, Taipei

## ﻿Results

### ﻿ Yufengus

Taxon classificationAnimaliaLepidopteraLimacodidae

Lin, Lin & Epstein gen. nov.

CF062DA8-F0D7-53C1-8859-7AAD62BFE05B

https://zoobank.org/FCB81D71-7956-4D9F-BAD6-85B9BD7BC7D5

#### Type species.

*Yufengus atrophaneuroides* sp. nov. (here designated).

#### Diagnosis.

No known adults or larvae of Limacodidae from Asia are similar in general appearance to *Yufengus*. Structurally they are most comparable to *Phrixolepia* as larvae (Figs 36–39, 52–56) and adults (Figs 57, 58), although adult *Phrixolepia* forewing lacks the distinct white forewing spots and dark subapical marking on ventral surface (Figs 9–12), have male filiform (Fig. 15) rather than bipectinate antenna (Fig. 13), have Rs4 originating beyond discal cell (Fig. 18) rather than from discal cell, have gnathos present (Fig. 21) rather than absent, have coiled ductus bursae (Fig. 22) rather than straight, and the labial palp 3^rd^ segment is much shorter compared to 2^nd^ segment compared to *Yufengus* (Fig. 16) (Note: the description of *Yufengus* is found below under *Y. atrophaneuroides*).

#### Etymology.

The genus is named after Dr Yu-Feng Hsu, the advisor of the first two authors, who has provided support and encouragement for limacodid studies over the past 10 years.

### ﻿ Yufengus atrophaneuroides

Taxon classificationAnimaliaLepidopteraLimacodidae

Lin, Lin & Epstein sp. nov.

A00AC1D9-AC61-50D1-993B-D9F47D652A8F

https://zoobank.org/D5DB4CBA-9BE3-49B0-BFB8-3749632A3D31

Figs 1–8, 13, 14, 17, 19, 20, 23–34, 41–51

#### Type materials.

**Figures 1–8.**
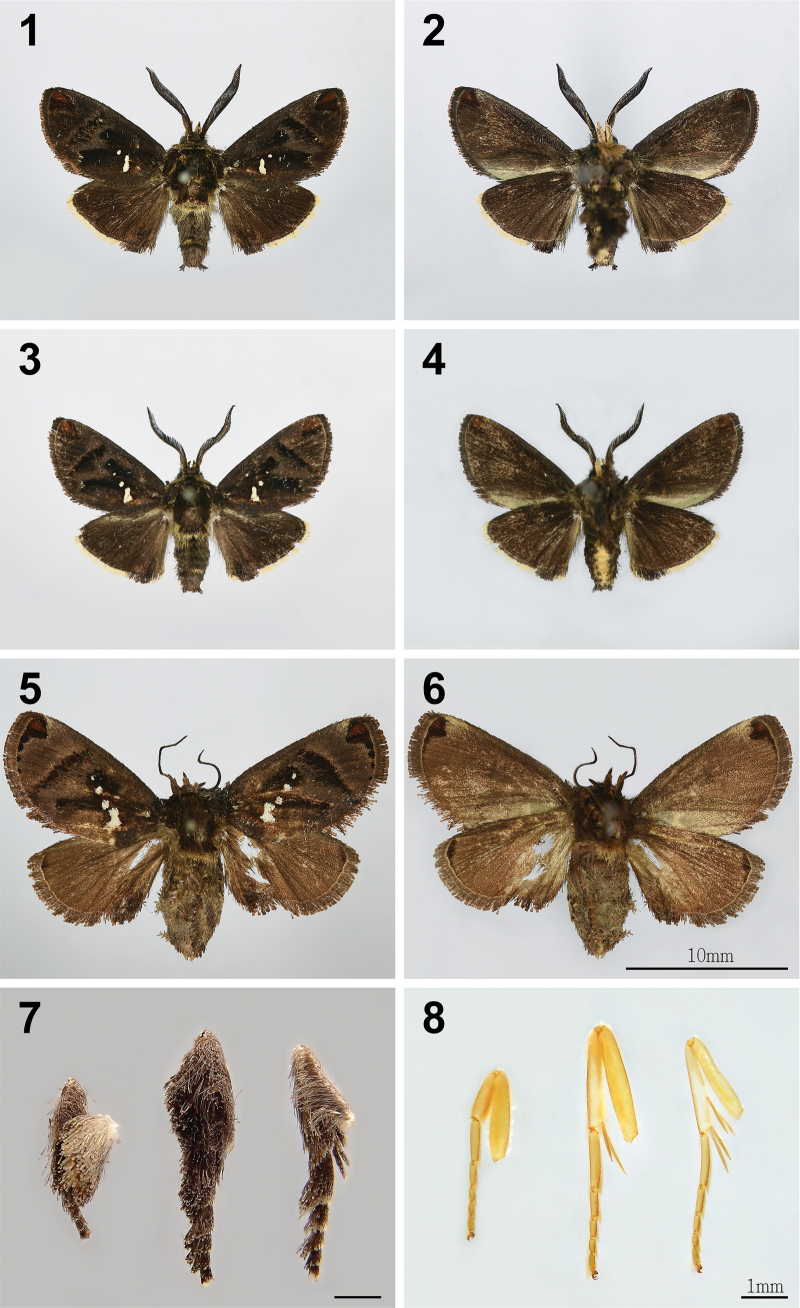
Adults of *Yufengus atrophaneuroides* sp. nov. **1, 2.** Male, holotype, HSU 22B39M, upperside and underside; **3, 4.** Male, paratype, HSU 22B40M, upperside and underside; **5, 6.** Female, paratype, HSU 16C67M, upperside and underside; **7, 8.** Male legs, paratype, HSU 22B39M, with scales and without scales (left to right: foreleg to hind leg). Scale bars: 10 mm (1–6), 1 mm (7, 8).

***Holotype*.** Taiwan • 1 ♂; New Taipei City, Wulai, Xinfu Rd.; 24.8259, 121.5240; alt. 380 m; 28 Feb. 2022; Y.C. Lin, R. J. Lin, C J. Hung & H. Chang leg.; reared from *Oreocnide pedunculata*, pupated 21 Mar. 2022, emerged 17 Apr. 2022; HSU 22B39M (Figs 1, 2).

***Paratypes*.** Taiwan • 1 ♂; New Taipei City, Wulai, Xinfu Rd.; 24.8259, 121.5240; alt. 380 m; 28 Feb. 2022; Y.C. Lin, R.J. Lin, C.J. Hung & H. Chang leg.; reared from *Oreocnide pedunculata*, pupated 25 Mar. 2022, emerged 23 Apr. 2022; HSU 22B39M (Figs 7, 8, 13, 14, 17, 19) • 1 ♂; New Taipei City, Wulai, Xinfu Rd.; 24.8259, 121.5240; alt. 380 m; 28 Feb. 2022; Y.C. Lin, R.J. Lin, C.J. Hung & H. Chang leg.; reared from *Mussaenda parviflora*, pupated 9 Mar. 2022, emerged 31 Mar. 2016; HSU 22B40M (Figs 3, 4) • 1 ♀; New Taipei City, Wulai, Xinxian; 24.8355, 121.5274; alt. 220 m; 31 Mar. 2016; M.X. Luo & H.P. Lu. leg.; reared from *Smilax bracteata*, emerged 4 May 2016; GenBank: MK128293; HSU 16C67M (Figs 5, 6, 20).

#### Diagnosis.

See under *Yufengus* above.

#### Description.

**Male** (Figs 1–8, 13, 14, 17). Head: frons hairy, brown with yellow scaling; eyes semioval, black; antenna bipectinate from base to apex, medial side of antenna protrusions shorter than the lateral side; labial palpus upcurved with the apex pointing dorsally, covering dark brown scales dorsally and yellow scales ventrally, with the 3^rd^ segment longer than 1/3 of 2^nd^ segment (Fig. 14); proboscis present (Fig. 13). Thorax: dark brown, with long, yellow hairs laterally and posteriorly near abdomen; legs brown, banded with yellow on tarsi (Fig. 7), midleg with one pair of tibial spurs, hindleg with two pairs of tibial spurs, each pair of unequal length (Fig. 8). Forewing: 8.16–8.79 mm long (*x̄*: 8.44 ± 0.32 mm, *n* = 3). Upperside ground color dark brown, a reddish-brown spot surrounded by black at apex, three black patches on discal cell, discal area, and postdiscal area. Four white spots from inner margin to discal cell (latter two sometimes invisible) (Figs 1, 3), fringe with dark-brown scales. Underside ground color dark brown, broadly pale grey along inner margin, a reddish-brown spot surrounded by black at apex. Forewing venation: Rs2 and Rs3 on common branch originating from Rs1 near discal cell; Rs4 originating from discal cell; MS bisecting discal cell; 1A+2A with basal fork. Hindwing: upperside dark brown; fringe with contrasting pale-yellow scales, becoming mostly black near tornus. Underside similar to upperside. Hindwing venation: MS present in discal cell; a very short cross vein between R and RS at the proximal third of the wing, inside the discal cell; RS and M1 meet at outer end of discal cell; CuP, 1A+2A, and 3A present as typical in Limacodidae. Abdomen: dark-brown dorsum, yellow in posterior half of ventrum.

**Figures 9–12.**
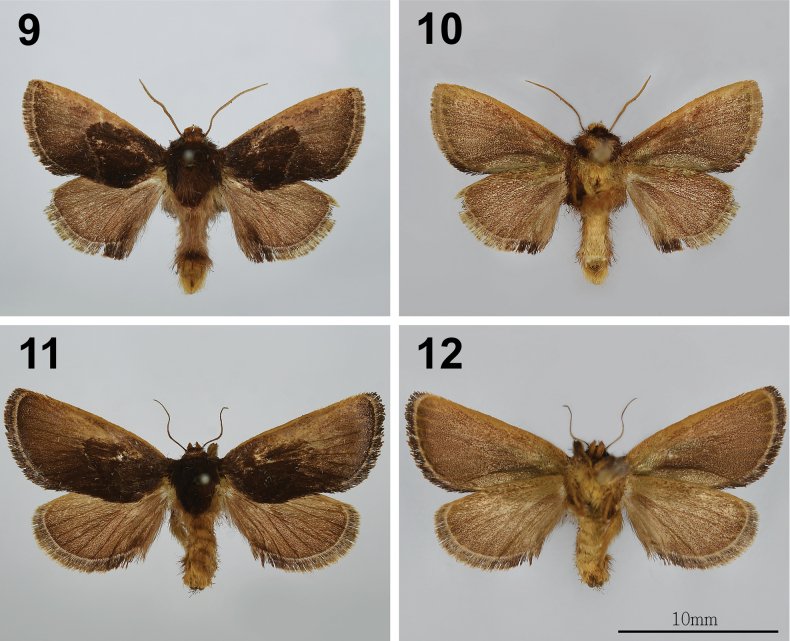
Adults of *Phrixolepia inouei*; **9, 10.** Male, upperside and underside; **11, 12.** Female, upperside and underside. Scale bar: 10 mm.

**Female** (Figs 5, 6, 50, 51). Similar to male, but larger (forewing 11.14 mm long, from base to apex); antenna filiform; ground color of wing brown, hindwing with a small dark-brown spot at apex, fringe of hindwing brown with dark scales throughout; the 5^th^ tarsomere with a triangular, recessed pad bearing sensilla and spinules (Fig. 51); abdomen brown ventrally.

***Male genitalia*** (Fig. 19). Uncus narrow throughout and curved slightly downward at apex. Gnathos absent. Tegumen and vinculum wider than the uncus. Juxta roughly triangular, slightly sclerotized, and setose. Valva elongate, round at apex and with short hairs on inner surface and slightly narrow at base; medial portion with sclerotized process with distal sawteeth. Saccus short. Phallus straight, tubular.

***Female genitalia*** (Fig. 20). Papillae anales flat, drop-shaped; anterior apophyses short, digitate, posterior apophyses about two times as long as anterior apophyses. Ductus burse membranous and straight. Corpus bursae ovate, about two-thirds length of ductus burse; signum with spines: two large and a few small ones.

**Figures 13–16.**
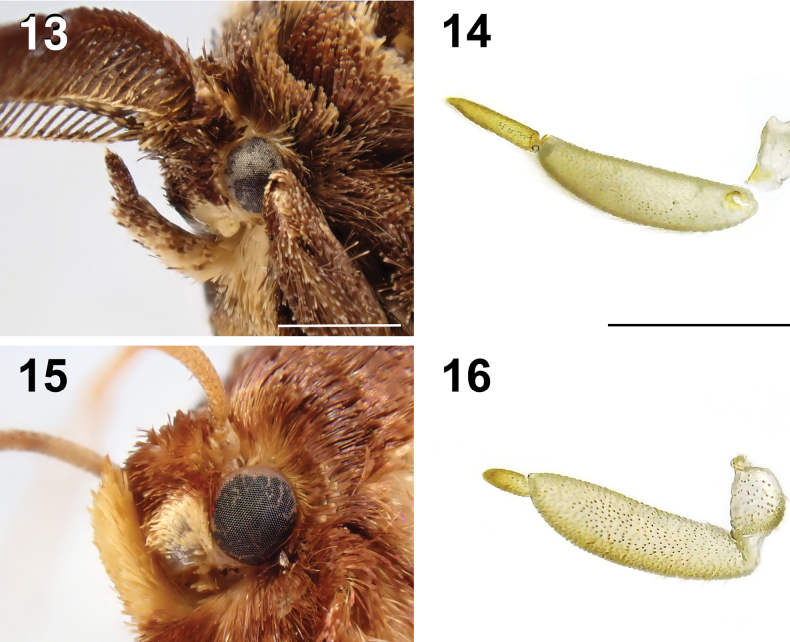
Adult head. **13.** Male head of *Yufengus atrophaneuroides* sp. nov. with only one side of labial palp and small proboscis, paratype, HSU 22B39M; **14.** Labial palp without scales of *Y. atrophaneuroides* sp. nov.; **15.** Male head of *P. inouei* with only one side of labial palp and small proboscis; **16.** Labial palp without scales of *P. inouei.* Scale bar: 1 mm.

***Immature stages*** (Figs 23–34, 41–49). Egg (Fig. 23): flat oval, 1.92–2.00 mm in long axis (1.97 ± 0.05 mm, *n* = 3) and 1.22–1.30 mm in short axis (1.26 ± 0.04 mm, *n* = 3). First instar larva (Fig. 24): yellow ground-colored body with broken orange stripes on mid-dorsum and dorsolateral lines. Prothorax (T1) without tubercles and T2 and T3 each have three tubercles on each side, one D tubercle on A1, two tubercles—D and SD—on A2–A9; each tubercle has three setae. Two L setae on each segment. Early to mid-instar larvae (Figs 25–28): ground color of body yellow to white; some individuals with black patches on the range of T3–A1 and A3–A5 (Fig. 26); some individuals in the stage before the final instar (Fig. 28) with the slightly visible color of the final instar. Final instar larva (Figs 29–32): 12.10–12.48 mm in length (12.3 ± 0.19 mm without spine length, *n* = 3), 4.93–6.69 mm in width of A3 (5.93 ± 0.9 mm without spine length, *n* = 3). Head dark brown; labrum with multiple spinules anteriorly and laterally (Fig. 42); stemmata with gaps of approximately the width of a stemma between stemma 1 and stemma 2, and between stemma 5 and stemma 6 (Fig. 43); has only one seta (encircled by stemmata); spinneret brush-like with apex tapered (Fig. 44). Body slug-like, ground color black. Prothorax (T1) white with no tubercles; mesothorax (T2) with two single black D tubercles, one single black SD tubercle, and two short white L tubercles; metathorax (T3) with a branched D tubercle (two black branched parts on a quadripartite base, Fig. 32), and two single black SD tubercles, and two short white L tubercles. First abdominal segment (A1) with only a branched D tubercle. Abdominal segments with same D tubercles as A2–A8 as A1 but have one single black SD tubercle on each segment, two short white outgrowths for L of A2–A8. The longest D tubercle is on A4 and there are red patches on the tubercle apices of A5–A8, especially distinct on A6 and A7; one long tubercle for D and one white tubercle for L of A9. All tubercles bear numerous plumose setae (Fig. 46) and a single long bristle with tactile function, and those for D and SD can be easily detached when touched. The pretarsal claw of the thoracic leg (Fig. 45) is wide proximally, hook-like distally, with hair-like setae. Cocoon (Figs 33, 34): spheroid, 8.36–6.80 mm in long axis (7.57 ± 0.78 mm, *n* = 3) and 6.96–5.63 mm in short axis (6.19 ± 0.69 mm, *n* = 3), brown, with silk blending setae of final instar and forming a thin outer membrane. Pupa (Figs 47–49): thin cuticle, pupal eyepiece with a fracture line, maxilla without maxillary extension, pupal frons rough and shagreened, dorsum of abdominal segments with patch of spicules along anterior margin.

**Figures 17, 18.**
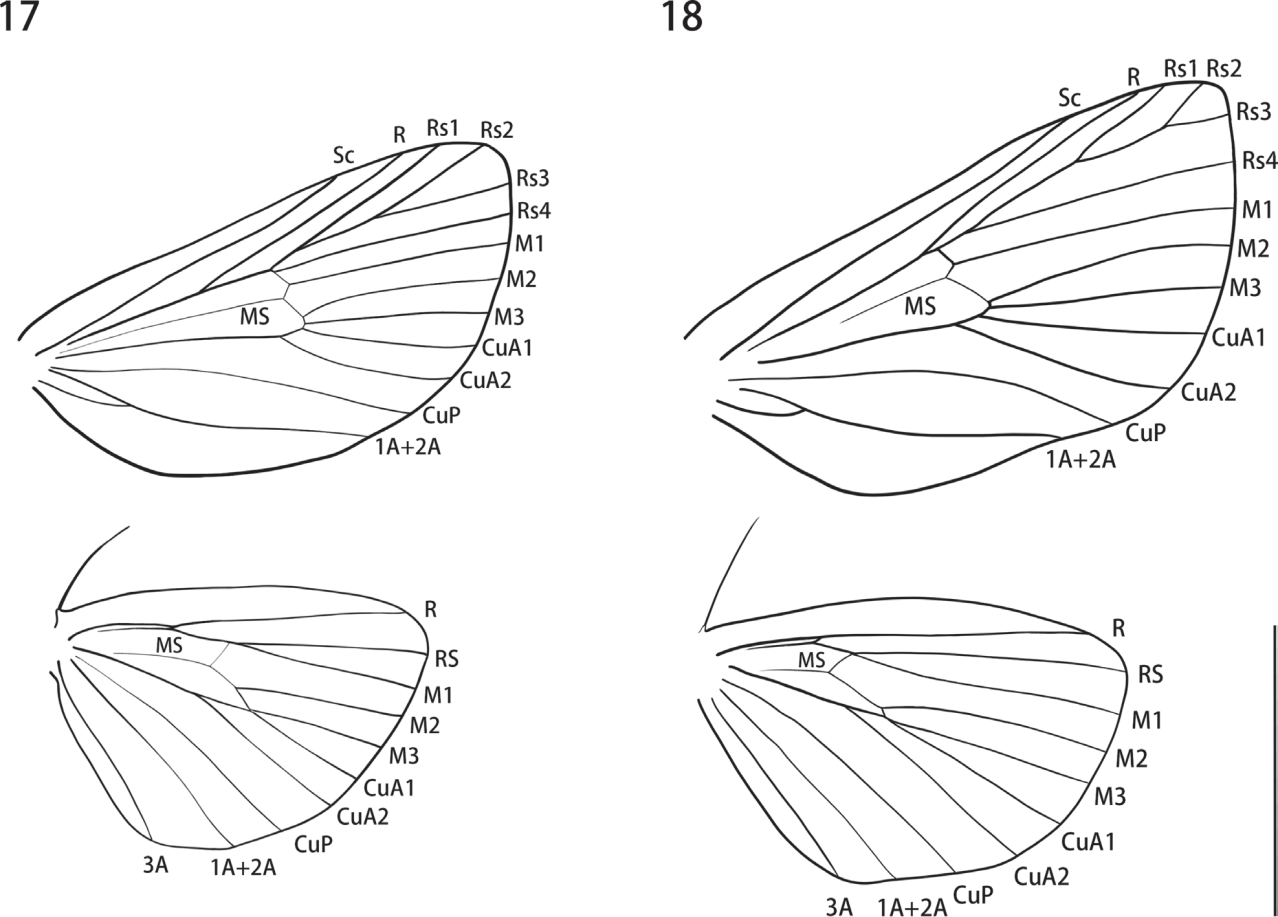
Wing venation. **17.***Yufengus atrophaneuroides* sp. nov., paratype, HSU 22B39M; **18.***Phrixolepia inouei.* Scale bar: 5 mm.

#### Etymology.

The specific name *atrophaneuroides* is derived from the swallowtail genus *Atrophaneura* Reakirt (Papillionidae) and the Latin suffix -*oides*, which means “-like”, because the black and red outgrowths of the final instar is reminiscent of *Atrophaneura* larvae. The species epithet is a compound descriptive name comprising an adjective combination with the ending of the second name in masculine form to agree in gender with the generic name.

**Figures 19–22.**
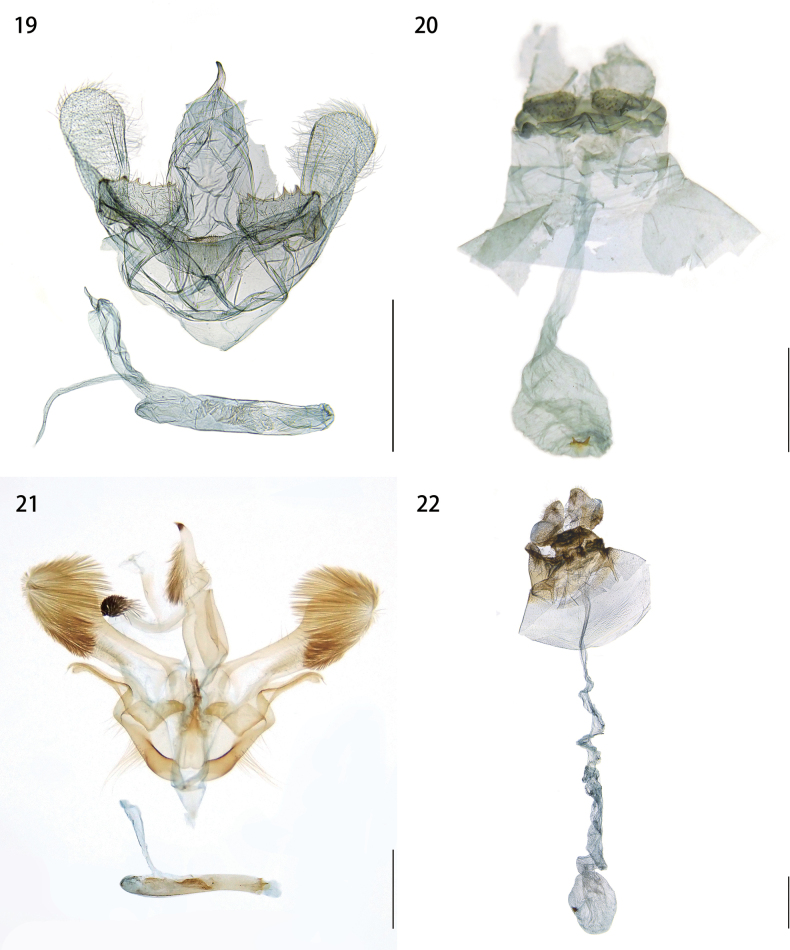
Genitalia. **19.***Yufengus atrophaneuroides* sp. nov., male, paratype, HSU 22B39M; **20.***Y. atrophaneuroides* sp. nov., female, paratype, HSU 16C67M; **21.***Phrixolepia inouei*, male; **22.***P. inouei*, female. Scale bar: 1 mm.

#### Distribution.

*Yufengus atrophaneuroides* is usually found from montane areas at low altitude in Taiwan.

#### Biology.

**Figures 23–40.**
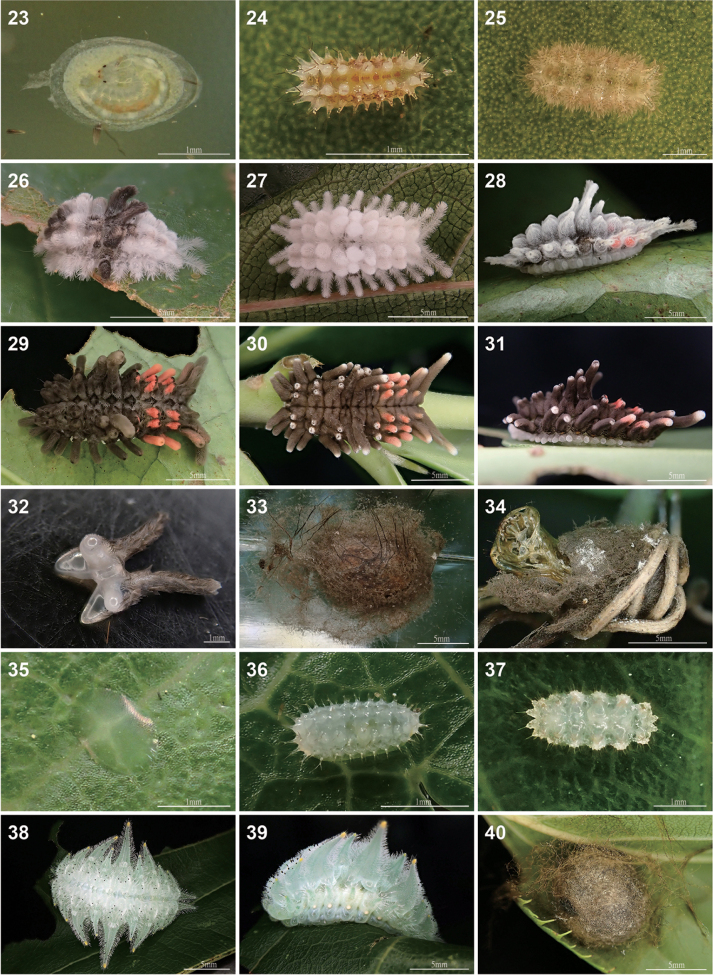
Immature stages of *Yufengus* and *Phrixolepia.***23, 34.***Yufengus atrophaneuroides* sp. nov.; **23.** Egg with late stage embryo; **24.** First instar; **25.** Second instar; **26–28.** Early instars; **29.** Final instar collected from New Taipei City, dorsal view; **30, 31.** Final instar collected from Hualien County, dorsal and lateral views; **32.** A quadripod tubercle of the final instar; **33, 34.** Cocoons with silk blending setae of final instar and forming a thin outer membrane; **35–40.***Phrixolepia inouei*; **35.** Egg; **36.** First instar; **37.** Second instar; **38, 39.** Final instar, dorsal and lateral views; **40.** Cocoon. Scale bars: 1 mm (**23–25, 32, 35–37**); 5 mm (**26–31, 33, 34, 38–40**).

*Yufengus atrophaneuroides* is polyphagous. Its larvae have been found on *Oreocnide pedunculata* (Shirai) Masam (Urticaceae) (HSU 22B39M), *Mussaenda parviflora* Miq. (Rubiaceae) (HSU 22B40M), Smilax bracteata C. Presl var. verruculosa (Merr.) T. Koyama (Smilacaceae) (HSU 16C67M, 22B42M), and Maesa perlaria (Lour.) Merr. var. formosana (Mez) Yuen P. Yang (Myrsinaceae) (HSU 22B41M). According to Taiwan Moth Information Center (https://twmoth.tbri.gov.tw), established and maintained by Taiwan Biodiversity Research Institute, *Yufengus* larvae have been found from January to June. Based on rearing data, larvae reared in captivity developed directly, with the pupal stage around a month. The cocoons (Fig. 33), similar to *Phrixolepia* (Fig. 40), do not encorporate the tubercles from the prepupa as in *Phobetron* (see figure in Epstein et al. in press). These observations suggest there are multiple generations of *Y. atrophaneuroides* annually. Eggs are laid singly (Fig. 23), similar to *Phrixolepia* (Fig. 35) and other gelatines (Epstein et al. in press).

#### Remarks.

The final instar larvae of *Y. atrophaneuroides* shows marked variation in tubercle apices from different locations. For example, there is no white patch on larvae collected from New Taipei City (Fig. 29); however, there are little white patches on larvae from Hualien County, eastern Taiwan (Figs 30, 31). According to the “Taiwan Moth Information Center”, these white patches are bigger on the larvae from Hsinchu County (collecting number: 497121; https://twmoth.tbri.gov.tw/peo/FBMothInfo/497121) than that from Hualien County; furthermore, the largest white patches occur on the larvae from Pingtung County (collecting numbers: 240751 and 339953; https://twmoth.tbri.gov.tw/peo/FBMothInfo/240751 and https://twmoth.tbri.gov.tw/peo/FBMothInfo/339953, respectively). Whether this represents discrete or continuous variation requires additional investigation. Another from Xinxian, Wulai, Taiwan on 15 Dec. 2021 by David Tai may be an earlier instar of *Y. atrophaneuroides* (https://twmoth.tbri.gov.tw/peo/FBMothInfo/466895).

The early instars of *Y. atrophaneuroides* closely resemble some mealybugs such as *Phenacoccus madeirensis* Green, 1923. Many mealybugs are covered with white, powdery or mealy wax secretion on the body, which are believed to protect them from predators (Gullan and Kosztarab 1997). According to a mealybug study in Taiwan (Tsai 2011), *P. madeirensis* is widely distributed and polyphagous, and it co-occurs with *Y. atrophaneuroides* in New Taipei city. In the field, mealybugs have also been found in the same area where *Y. atrophaneuroides* occurs. Hence, the possibility of mimicry cannot be excluded.

There may be additional species in *Yufengus*, as suggested by larval photographs available online from outside Taiwan. For example, one is observed in Gunung Mulu National Park, Sarawak, Malaysia, at 131 m on 29 Sept. 2012 by Bernard Dupont (https://www.inaturalist.org/observations/85512375).

## ﻿Discussion

Although sampling of taxa differs between Lin et al. (2019) and Epstein et al. (in press), these studies suggest a relationship between *Phrixolepia* with the *Phobetron* complex or a genus within it, the *Prolimacodes* complex, or both complexes. As mentioned above, Lin et al. (2019) suggested *Yufengus* was related to *Phrixolepia* and *Isochaetes.* In total evidence analysis, Epstein et al. (in press) found *Phrixolepia* to be sister to the *Phobetron* complex (including *Isochaetes*) + the *Prolimacodes* complex but with molecular analysis alone (ML and BI) found *Phrixolepia* to be sister to the *Prolimacodes* complex.

**Figures 41–58.**
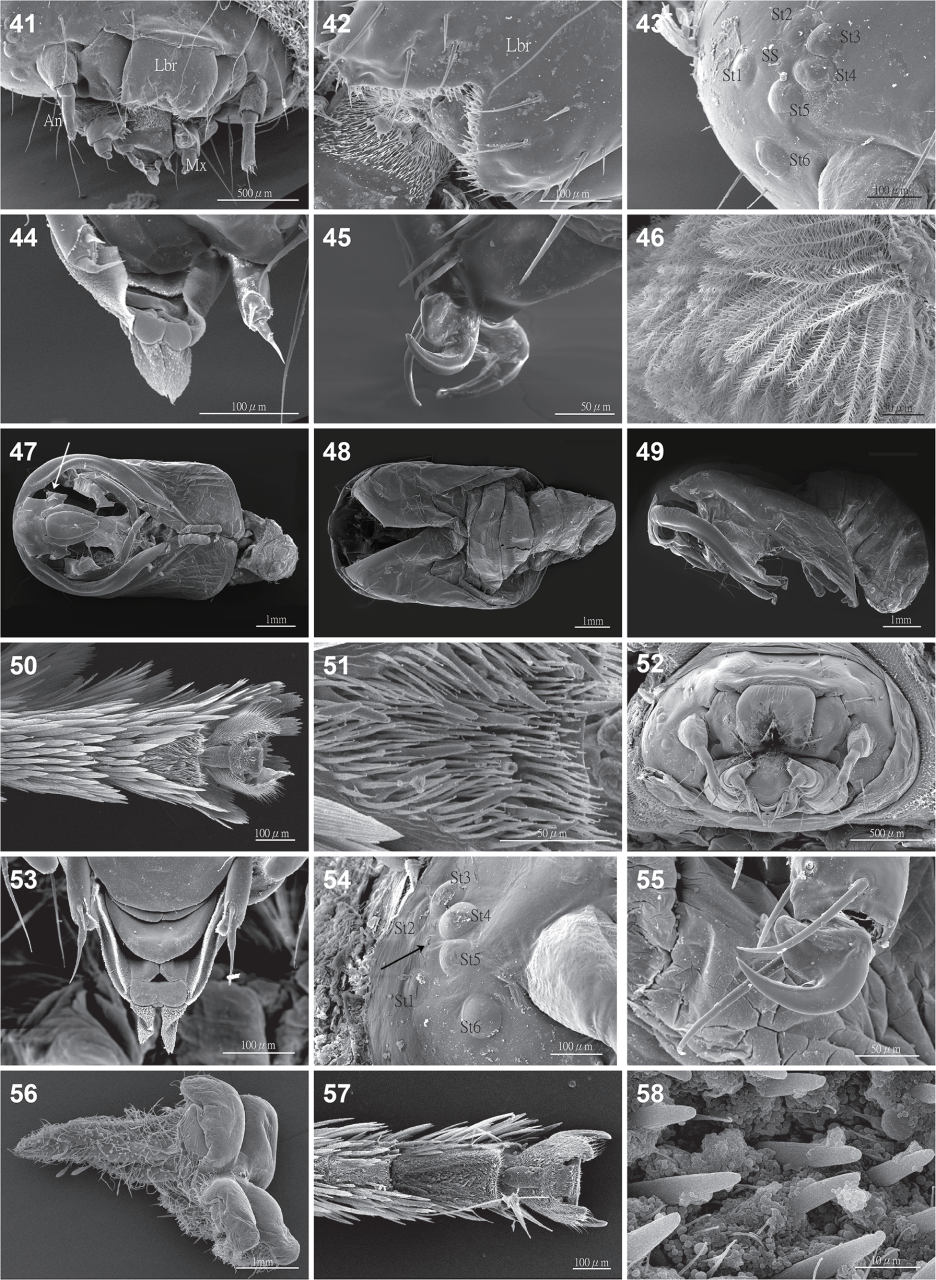
The final instar larva, pupa, and female 5^th^ tarsomere by scanning electron microscope. **41–51.***Yufengus atrophaneuroides* sp. nov.; **41.** Larval head (Lbr: labrum, An: antenna, Mx:maxillary palps; **42.** Larval labrum with multiple spinules anteriorly and laterally; **43.** 6 stemmata (St1–6) and stemma seta (SS); **44.** Spinneret; **45.** Pretarsal claw of the thoracic leg without axial seta; **46.** Plumose setae of larval tubercles; **47–49.** Eclosed pupa (arrow points to the location of absent maxillary extension), ventral, dorsal, and lateral views; **50.** Female 5^th^ tarsomere with sensilla trichodea; **52–58.***Phrixolepia inouei*; **51.** Sensilla and spinules of female 5^th^ tarsomere; **52.** Larval head; **53.** Spinneret; **54.** 6 stemmata and stemma seta (arrow points to the remaining seta pit); **55.** Pretarsal claw of the thoracic leg with axial seta; **56.** A quadripod tubercle of the final instar; **57.** Female 5^th^ tarsomere with sensilla trichodea; **58.** Sensilla of female 5^th^ tarsomere. Scale bars: 500 μm (**41, 52**); 100 μm (**42–44, 50, 53, 54, 57**); 50 μm (**45–46, 51, 55**); 1 mm (**47–49, 56**); 10 μm (**58**).

Even though *Yufengus* has differences with *Phrixolepia* or *Isochaetes* as larva or adult, the remainder of this section we will explore their similarities to these and other genera with deciduous tubercles including New World *Phobetron* complex and Asian *Olona* Snellen.

Along with sharing deciduous tubercles there are other larval similarities between *Yufengus atrophaneuroides*, the New World *Phobetron* and *Prolimacodes* complexes, and *Phrixolepia.* These include a quadripartite tubercle base, branched D tubercles, and the absence of an SD tubercle on A1. Note that *Olona* also has a quadripartite tubercle base along with deciduous tubercles (Solovyev et al. 2012). An important difference between the larval morphology of *Y. atrophaneuroides* and the others is the appearance of “extra” tubercles after the first instar.

Additional larval similarities shared between *Yufengus* and *Phrixolepia* are also found in both the New World *Phobetron* and *Prolimacodes* complexes. These include a v-shaped spinneret in late instar (see Epstein 1996; Epstein et al. in press). Pupal similarities include the absence of a maxillary extension (Fig. 47).

Adult similarities shared between *Yufengus* and the New World genera *Phobetron*, *Isochaetes*, *Semyra*, and *Prolimacodes* include a branching Rs2 + Rs3 off of Rs1, a straight aedeagus, and the dorsal medial process of the valvae. Adults of *Yufengus* are similar to *Semyra* in two aspects of the forewing: the silver macula below the discal cell on the dorsum and a brown patch near the apex of the ventrum.

In conclusion, the retention of tubercles in *Yufengus* and others, albeit deciduously, is not so odd given that most other gelatines, including the *Prolimacodes* complex, do not possess tubercles after the first instar. It is for future research with more taxon sampling to determine a more precise placement for *Yufengus* and *Phrixolepia*, and other Old World gelatines with or without deciduous tubercles.

## Supplementary Material

XML Treatment for Yufengus

XML Treatment for Yufengus atrophaneuroides

## ﻿Additional information

### Author ORCIDs

Yu-Chi Lin https://orcid.org/0000-0001-5615-0188

Marc E. Epstein https://orcid.org/0000-0001-8659-5815

### Data availability

All of the data that support the findings of this study are available in the main text.
